# Stacking multiple connecting functional materials in tandem organic light-emitting diodes

**DOI:** 10.1038/srep43130

**Published:** 2017-02-22

**Authors:** Tao Zhang, Deng-Ke Wang, Nan Jiang, Zheng-Hong Lu

**Affiliations:** 1 Department of Physics, Yunnan University, Kunming, Yunnan 650091, People’s Republic of China; 2 Yunnan Key Laboratory for Micro/Nano Materials and Technology, Yunnan University, Kunming, Yunnan 650091, People’s Republic of China; 3 Department of Materials Science and Engineering, University of Toronto, Toronto, Ontario M5S 3E4, Canada

## Abstract

Tandem device is an important architecture in fabricating high performance organic light-emitting diodes and organic photovoltaic cells. The key element in making a high performance tandem device is the connecting materials stack, which plays an important role in electric field distribution, charge generation and charge injection. For a tandem organic light-emitting diode (OLED) with a simple Liq/Al/MoO_3_ stack, we discovered that there is a significant current lateral spreading causing light emission over an extremely large area outside the OLED pixel when the Al thickness exceeds 2 nm. This spread light emission, caused by an inductive electric field over one of the device unit, limits one’s ability to fabricate high performance tandem devices. To resolve this issue, a new connecting materials stack with a C_60_ fullerene buffer layer is reported. This new structure permits optimization of the Al metal layer in the connecting stack and thus enables us to fabricate an efficient tandem OLED having a high 155.6 cd/A current efficiency and a low roll-off (or droop) in current efficiency.

As compared with conventional organic light-emitting diodes (OLEDs), tandem OLEDs have received a broad attention owning to their superior current efficiency, brightness and operational lifetime^1^,^2^,^3^,^4^,^5^,^6^,^7^. In tandem OLEDs, several individual electroluminescent (EL) units are electrically connected in series via connecting stacks (sometime referred to as connecting electrodes) which function as charge generation layer (CGL), where holes and electrons are generated and injected into the adjacent hole transporting layers (HTL) and electron transporting layers (ETL), respectively. In principle, the device characteristics such as voltage, luminance, and current efficiency scale linearly with the number of EL units for tandem device with an efficient CGL^8^,^9^,^10^,^11^,^12^,^13^,^14^,^15^,^16^,^17^.

Recently, various efforts have been devoted to the development of high performance CGL structures, which have a “p-n junction” characteristic. For instance, the combination of the transition metal oxide (TMO) such as molybdenum trioxide (MoO_3_) and HTL is widely used as p-type layers due to the very low conduction band minimum (CBM) and the high work function^10^,^11^,^12^,^13^,^14^,^15^,^18^. As for n-type CGL materials, an ultrathin bilayer Liq/Al is used as an electron extraction layer^8^,^16^,^17^,^18^. For a typical organic/metal/metal oxides electrode system, TMO serves as a strong electron acceptor, when electrons are drawn from the highest occupied molecular orbital (HOMO) of HTL to the CBM of TMO, and thus electron-hole pairs are generated.

In this paper, we reports experimental findings of a leaky lateral current spreading over connecting electrodes when the Al thickness is increased to more than 2 nm. This type of leaky connecting electrodes induces electric field over one of the device unit, and causes a spread light emission over an extremely large area outside the OLED pixel. This lateral current spreading seriously limits one’s ability to maximize the full potential of tandem OLEDs. To solve this problem, we report a new connecting materials stack Liq/Al/C_60_/CBP:MoO_3_ which enables tandem OLEDs having a high current efficiency of 155.6 cd/A.

## Considerations for Constructing a Metal-containing CGL

**Figure 1:**
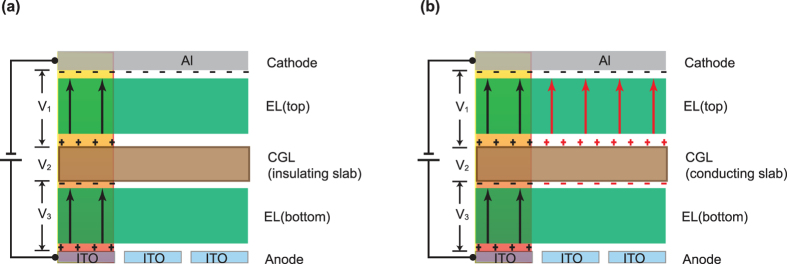
Schematic illustration of a tandem device with (**a**) a CGL having no lateral electrical conduction and (**b**) a CGL having lateral electrical conduction.

For a conventional OLED, the semi-insulating organic semiconductor is sandwiched between two electrodes, forming a pixel plate capacitor^19^. A capacitor can induce charges on the two inner surfaces of the metal plates when biased. For a typical tandem OLED, a metal-containing connecting electrode stack is inserted between these two electrodes. Under forward bias, electric field will induce surface charges on these various electrode surfaces, as illustrated in Fig. 1.

As shown in Fig. 1a, the total applied voltage V between the anode and cathode plates can be approximated as:


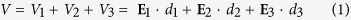


where **E**_**1**_ and d_1_ are the electric field strength and the distance between anode and CGL, **E**_**2**_ and d_2_ are the electric field strength and the distance in CGL, **E**_**3**_ and d_3_ are the electric field strength and the distance between CGL and cathode. As indicated in Equation (1), it is important to voltage loss (V_2_) to the connecting stacks in order to reduce the device operating voltage (V). So it is rationale to decrease V_2_ by increasing the thickness of the metal layer in connecting stacks. As will be shown in the following text, however, a simple increase in metal layer thickness in connecting stacks will result in an increase in lateral conductivity and thus leads to an induced electric field extended far beyond the pixelated region, as shown in Fig. 1b. Therefore, it is important to design and construct a CGL stack that has minimum voltage loss and yet has no lateral conduction.

To assist quantification of device performance, an optical model is used to simulate two separate emission zones in a tandem device. The optical electric vector and the relative spectral power distribution of tandem OLEDs are calculated by using a dipole source term and transfer matrix approach^20^. Exciton distribution and weak microcavity effect^21^ in a tandem OLED are also considered in device modeling.

Organic molecular emission process is considered as spontaneous radiation. The emitted photons from the two separate emission zones are mutually incoherent. Based on this assumption, the total spectral power distribution *P(λ*) of tandem device can be obtained by the sum of normalized spectral power distribution from each emission zone (details are provided in Supplementary Information). Each emission zone’s spectral power distribution in the same device is calculated separately, and the photoluminescence (PL) spectrum of Ir(ppy)_2_(acac) is used as the emission source.

The theoretical current efficiency of a tandem device in the normal direction is calculated by the following equation:


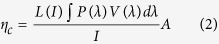


where *V(λ*) is the normalized photopic spectral response function; *L(I*) is the luminous intensity at injection current *I* which is the measured experimental data; A is the device active area.

The angular luminance distribution is simulated by:


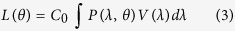


where *P(λ, θ*) is the relative spectral power distribution at a viewing angle *θ; C*_0_ is a constant (683 lm/W).

## Results and Discussion

**Figure 2:**
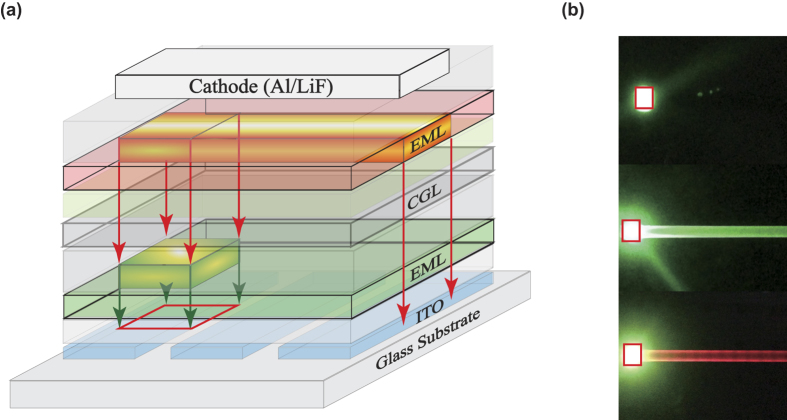
(**a**) Schematic device structure. (**b**) photograph picture of the device-A with 1 nm Al layer (top panel); photograph picture of the device-A with 2 nm Al layer (mid panel); photograph picture of the device-B with 2 nm Al layer (bottom panel).

**Figure 3:**
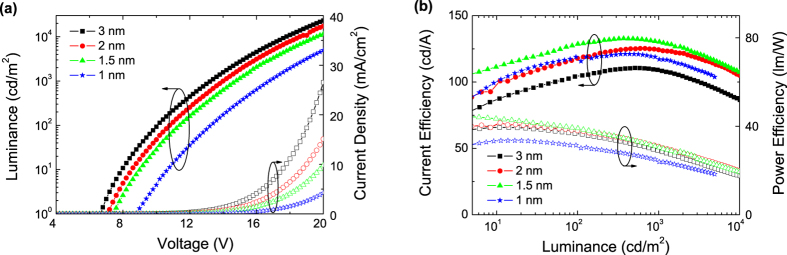
(**a**) L–I–V, (**b**) current and power efficiency-luminance characteristics of device-A with various thicknesses of Al layer.

The device structures of the tandem and corresponding single-unit OLEDs are summarized in Table 1. The schematic diagram of the tandem device structure and photographs of the devices with various thicknesses of the intermediate Al layer in the CGL are shown in Fig. 2. Based on above discussions, Fig. 3 illustrates the luminance–current–voltage (*L–I–V*) and the current-power efficiency characteristics of a set of devices using the device-A structure. As shown in Fig. 3a, the turn-on voltage decreases whereas the current density increases as the Al layer thickness increases. This indicates that a thicker Al layer is desired for reduction of device driving voltages, as expected based on the above discussion. The maximum current and power efficiencies of this device with a simple organic/metal/TMO stack are 132.8 cd/A and 44.6 lm/W, achieved with 1.5 nm Al layer. From operating voltage point of view, Fig. 3a clearly shows the benefit of thicker Al layer. The device performance of these tandem devices are found in good agreement with optical modeling shown as Supplementary Fig. S1.

**Figure 4:**
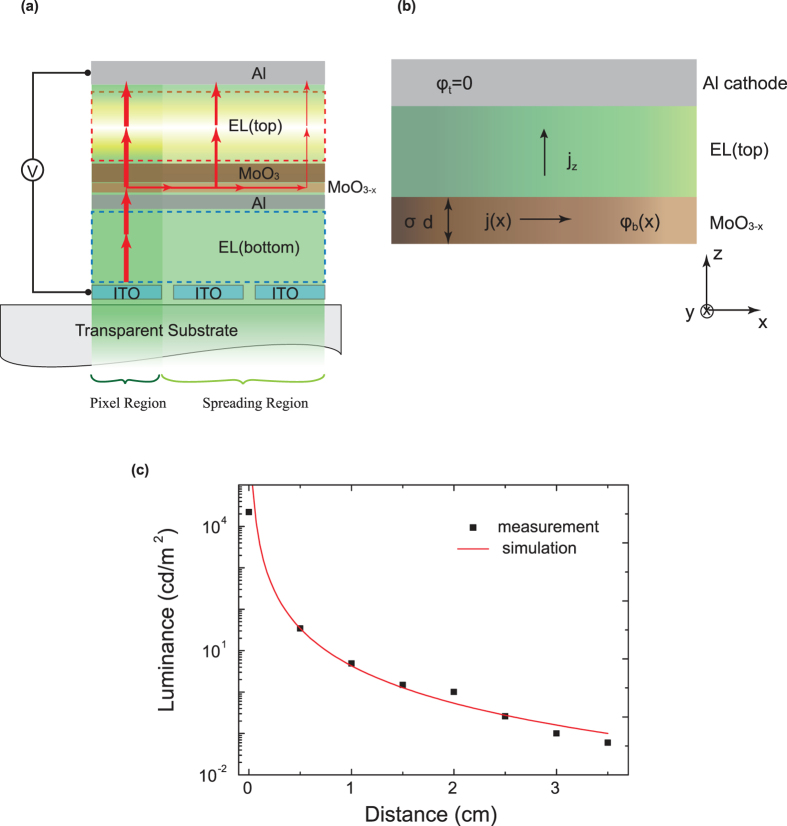
(**a**) The working mechanism model of the tandem device-A with Liq/Al/MoO_3_, the MoO_3–x_ denote the oxygen-deficient MoO_3_, the arrows denote the current direction in the working device. (**b**) Cross-section of OLED device with Al cathode and intermediate metallic or semimetallic material MoO_3–x_ layer as bottom electrode. (**c**) Comparison of measurement and simulation for the luminance - distance characteristic for device-A with 2 nm Al.

Surprisingly, as the Al thickness is increased to more than 2 nm, there is a light emission spread over a large area outside the OLED pixel (Fig. 2b mid panel). This suggests that there is an induced electric field extended far beyond the pixelated region and a lateral current spreading over the connecting electrode when the Al thickness exceeds 2 nm. To identify the light-emission zone of the spread emission, a test tandem device-B with red (top unit) and green emission (bottom unit) units was fabricated. The photograph picture of light spreading from device-B is shown in Fig. 2b bottom panel. The red color of the spread light outside the pixel indicates that the light-emission zone originates from the top EL unit. The use of a thicker Al layer apparently increases the vertical as well lateral electrical conductivity. The light spreading is explained as due to a lateral current spreading in the CGL, as shown schematically in Fig. 4.

Based on our previous research that metal contacts will affect the interface MoO_3_’s chemical and electronic properties, the metal-metal oxides interfacial reaction will lead to changes in the MoO_3_’s chemical structure and energy band structure^22^. It is known that Al is a reactive metal, which can be very easily oxidized upon contact with MoO_3_. This causes Mo^6+^ cations reduced to lower oxidation states (Mo^5+^ and Mo^4+^) at the Al/MoO_3_ interface region. This interfacial oxide MoO_3–x_ contains Mo^5+^ and Mo^4+^ cations, which are metallic or semimetallic^22^,^23^,^24^. This reduction reaction leads to a conductive interfacial layer and thus a lateral current spreading along the MoO_3–x_ layer, which subsequently leads to a spread light emission along the top Al cathode. To quantify the light-spreading of the device-A, Fig. 4c plots the luminance vs distance from the edge of the pixel. It is noted that Fig. 2b shows a non-uniform emission along the cathode line. A non-uniform brightness in the OLEDs is possibly caused by a high electrode resistivity as discussed in a previous paper^25^. Here, the relatively low conductivity of the MoO_3–x_ is believed to be the cause of the inhomogeneous brightness. The relatively low conductivity of the MoO_3–x_ also leads to an appreciable lateral voltage drop in the connecting electrode stack. This also results in a luminance drop over the spread light emission region.

To model the luminance-distance experimental data, we consider the OLED as three layers (Fig. 4b): a MoO_3–x_ layer (bottom electrode), an emitting layer (top EL), and a top Al cathode. The thicknesses of these layers and the dimensions of the y coordinate are much smaller than the lateral dimensions of the x coordinate of the OLED device. In the model we can make the following approximations:

1. The cathode can be considered as a perfect conductor and it is grounded, φ_t_ = 0;
2. The potential in the MoO_3–x_ layer is practically independent of the y coordinate and z coordinate, φ_b_(x);
3. The current density in the emitting layer is perpendicular to the substrate, j_z_

The luminance L scales linearly with the current density and assuming the device efficiency η is constant. Thus


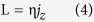


The lateral current density in the MoO_3–x_ layer is proportional to the electric field and the material conductivity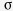. This can be expressed as^25^,


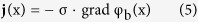


The conservation of the current flux in a steady state requires^25^


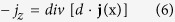


These equations lead to that the luminance is related to the distance in an inverse cubic relationship. The simulated luminance as a function of the distance is shown in Fig. 4c, which shows a very good agreement with the experimental data.

As a side note, the Al/MoO_3_ structure is also widely used in tandem organic solar cells^26^,^27^. Based on the above findings on current spreading, it is extremely important to take the current spreading into account so that the power conversion efficiency from a tandem solar cell may be calculated more accurately.

To eliminate the current spreading, one way is to reduce the thickness of the MoO_3_ layer to below 3 nm. The efficiency of the device, however, cannot be improved by a simple optimization in the metal oxides thickness (see Supplementary Fig. S2). Another way is replacing the MoO_3_ to CBP:MoO_3_ layer. In order to maximize the efficiencies of tandem devices, devices C-F using various kinds of CGL were fabricated. Because of its high electron mobility and strong electron accepting ability, a C_60_ buffer layer is introduced to prevent oxidation reduction reaction at the Al-MoO_3_ interface. This buffer layer will reduce the device driving voltage and thus enhance the device efficiency. In order to examine the impact of C_60_ insertion on CGL, we have made a test device structure with embedded finger electrodes to measure the lateral conductance. The measurement shows that the lateral electric conductivity of the CGL without a C_60_ layer is 5.25–31.50 S/cm, while the lateral electric conductivity of the CGL with a C_60_ layer is 0.41–2.44 S/cm (details are provided in Supplementary Information). This confirms that the inserting of C_60_ layer between Al and MoO_3_ layers can effectively suppress the lateral conductivity of the CGL. To eliminate possible run-to-run variations, tandem OLED devices were fabricated on the same substrate by changing shadow masks. For comparison, a reference device with a single EL unit was also prepared. The detailed layer structures of the devices are shown in Table 1.

**Figure 5:**
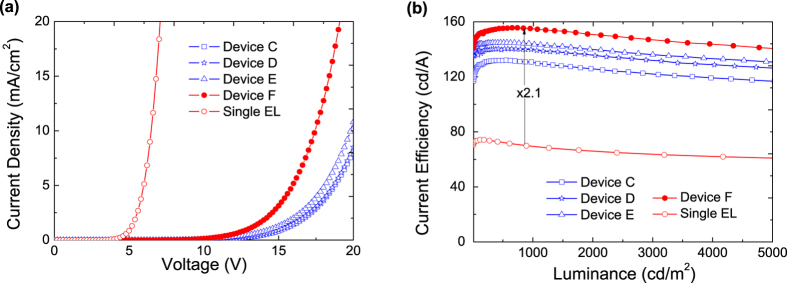
(**a**) Current density–voltage (J-V), (**b**) Current efficiency–luminance characteristics of single EL unit device and tandem devices C-F with different CGL. The device configurations are shown in Table 1.

The current density - voltage (J-V), and current efficiency - luminance (CE–L) characteristics of four tandem OLEDs (devices C-F) together with that of a single EL unit device are shown in Fig. 5. A significantly increased current density is observed for device-F as shown in Fig. 5a. This indicates that a thicker Al layer can effectively enhances charge injection properties of the Liq/Al/C_60_/CBP:MoO_3_-based tandem OLEDs. As shown in Fig. 5b, the device efficiencies of device-E (Liq/Al/C_60_/CBP:MoO_3_) is higher than device-D (Liq/Al/C_60_/MoO_3_), this can be attributed to the smaller hole injection energy barrier. It is well known that by effective doping method^28^,^29^, the Fermi level within the MoO_3_ doped CBP is close to the HOMO level of CBP. While the C_60_ contact with CBP:MoO_3_ layer, a common Fermi level throughout both layers is required by the equilibrium, which is close to the HOMO of CBP:MoO_3_. Therefore, under the applied electrical field, the electrons may tunnel through from HOMO of CBP:MoO_3_ to the lowest unoccupied molecular orbital (LUMO) of C_60_, then these electrons will immediately be driven away from the interface and injected by the Liq/Al into the adjacent TPBi layer, and holes would transport in opposite directions to adjacent CBP layer simultaneously. Among these devices, device-F showed a current efficiency of 155.6 cd/A, which is much higher than that of others tandem OLEDs. Moreover, device-F also exhibits a low efficiency droop or roll-off. The current efficiency remains as high as 140.1 cd/A at a high luminance of 5000 cd/m^2^.

**Figure 6:**
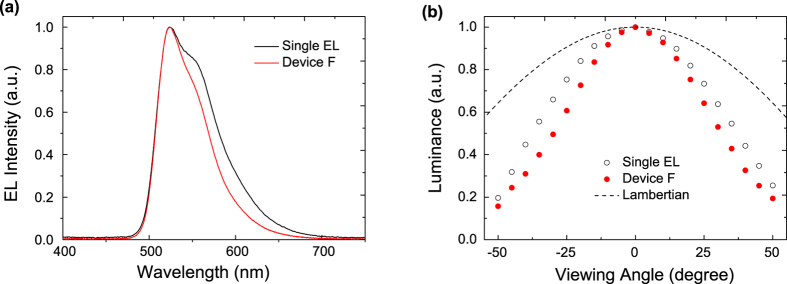
(**a**) EL spectra, (**b**) Measured and Lambertian (dashed line) angular distributions of the luminance (normalized to 0° intensity) for the single EL unit device and device-F.

According to the results, the current efficiency of the tandem device-F is more than twice higher than that of the single EL unit device. To understand this observation, the EL spectra and the luminance as a function of the viewing angle for the single EL unit device and device-F are measured and shown in Fig. 6. As shown in Fig. 6a, the tandem device-F shows a narrowing of the EL spectra as compared with the EL spectra from single unit device. This variation in the EL spectra is due to a microcavity effect in the devices and is in good agreement with optical modeling shown in Supplementary Fig. S3. The angular light distribution of both the single EL unit device and device-F are narrower than a Lambertian function as shown in Fig. 6b. It can clearly be seen that device-F has a narrower angular distribution and thus a stronger microcavity effect as compared with a single EL device. The doubling current efficiency of the tandem device-F is simply related to optical cavity effect. A theoretical optical model of the single EL device and device-F also shows characteristics similar to experimental data (see Supplementary Fig. S3).

Although an anti-oxidant C_60_ buffer layer is introduced to prevent oxidation reduction reaction at the Al-MoO_3_ interface, there is still a significant current lateral spreading causing light emission extended over a large area outside the OLED pixel when the Al thickness in the CGL exceeds 5 nm. This can be attributed to the formation of a continuous and laterally conducting Al film and thus resulting in an induced electric field extended far beyond the pixelated region. The device efficiencies with various thicknesses of the intermediate Al layer in the CGL (Liq/Al/C_60_/CBP:MoO_3_) are shown in Supplementary Fig. S4. A 3 nm Al layer is found to yield the best tandem device efficiency.

## Conclusions

In summary, we discovered that there is a significant current lateral spreading over the connecting electrode causing light emission extended over a large area outside the tandem OLED pixel when the Al thickness in the connecting electrode exceeds 2 nm. This current spreading limits the use of thicker Al and thus prevents optimization of the connecting stack for constructing super high performance tandem devices. In order to solve this problem, an anti-oxidant C_60_ buffer layer is introduced to prevent oxidation reduction reaction at the Al-MoO_3_ interface and to reduce the device driving voltage. This buffer layer enables us to optimize various functional materials in the connecting materials stack so that a high performance tandem OLED can be made.

## Materials and Methods

### Materials and Device Fabrication

In our work, all devices were fabricated in a tri-chamber high-vacuum thermal evaporation system with a base pressure of ~10^−7^ Torr on patterned indium tin oxide (ITO) coated glasses with a sheet resistance of 15 Ω/□. The active area of each device is 2 mm^2^. The tandem OLEDs were fabricated with two individual EL units, which were separated by a connecting materials stack or CGL. The EL-G (G stands for green color) consists of an HTL of 4,4′-Bis(carbazol-9-yl)biphenyl (CBP), an emitting-layer (EML) of Bis(2-phenylpyridine)(acetylacetonate)iridium (III) (Ir(ppy)_2_(acac)) doped with CBP and an ETL of 1,3,5-tris(N-phenylbenzimiazole-2-yl)benzene (TPBi), while the EL-R (R stands for red color) consists of an HTL of CBP, an EML of Bis(1-phenylisoquinoline)(acetylacetonate)iridium(III) (Ir(piq)_2_(acac)) doped with CBP and an ETL of TPBi. Bi-layer LiF/Al and MoO_3_/ITO were chosen as the cathode and anode, respectively. These materials were purchased from Luminescence Technology Corporation and used in as-received form for device fabrication. The detailed layer structures and thickness of all the devices or units in this work are shown in Table 1.

The deposition rate was 1.0 Å/s for organics and Al layer in the CGL, and was 0.1 Å/s for LiF, Liq and MoO_3_. Finally, Al cathode was evaporated on LiF film at a rate of 2 Å/s. The substrates were cleaned following a standard cleaning procedure^30^. All the organic layers were deposited in an organic chamber. The cathode was deposited in a separate metal chamber. The thickness of each layer was measured by a quartz crystal microbalance which had been calibrated using a spectroscopic ellipsometer.

### Device Measurement

The current–voltage (*I*–*V*) characteristics were measured using a HP4140B picoammeter. The luminance–voltage (*L*–*V*) measurements were performed using a Minolta LS–110 Luminance meter. The EL spectra were measured using an Ocean Optics USB4000 spectrometer. All measurements were carried out in ambient atmosphere at room temperature.

## Additional Information

**How to cite this article**: Zhang, T. *et al*. Stacking multiple connecting functional materials in tandem organic light-emitting diodes. *Sci. Rep.* **7**, 43130; doi: 10.1038/srep43130 (2017).

**Publisher's note:** Springer Nature remains neutral with regard to jurisdictional claims in published maps and institutional affiliations.

## Supplementary Material

Supplementary Information

## Figures and Tables

**Table 1:** Structures of the various test OLED devices.

Device ID	Unit One	CGL	Unit Two
Device A	EL-G	Liq(1 nm)/Al(x=1–3 nm)/MoO_3_(10 nm)	EL-G
Device B		Liq(1 nm)/Al(2 nm)/MoO_3_(10 nm)	EL-R
Device C		Liq(1 nm)/Al(1.5 nm)/MoO_3_ (10 nm)	EL-G
Device D		Liq(1 nm)/Al(1.5 nm)/C_60_(2 nm)/MoO_3_(10 nm)	
Device E		Liq(1 nm)/Al(1.5 nm)/C_60_(2 nm)/CBP:MoO_3_(50 wt.%, 10 nm)	
Device F		Liq(1 nm)/Al(3 nm)/C_60_(2 nm)/CBP:MoO_3_(50 wt.%, 10 nm)	
Single EL	ITO/MoO_3_(1 nm)/EL-G/LiF(1 nm)/Al(100 nm)		
EL-G	CBP(20 nm)/CBP:Ir(ppy)_2_(acac)(8 wt.%, 30 nm)/TPBi (65 nm)		
EL-R	CBP(20 nm)/CBP:Ir(piq)_2_(acac)(5 wt.%, 30 nm)/TPBi(65 nm)		

The general structure is ITO/MoO_3_(1 nm)/Unit One/CGL/Unit Two/LiF(1 nm)/Al(100 nm).
