# Supplementary material for: Stacking multiple connecting functional materials in tandem organic light-emitting diodes
Source: Sci Rep. 2017 Feb 22;7:43130. doi: 10.1038/srep43130 (PMC5320488; doi:10.1038/srep43130)
Supplement: Supplementary Information [file srep43130-s1.pdf]

Supplementary Information

Stacking multiple connecting functional materials in tandem organic light-emitting diodes

*Tao Zhang[1,2], Deng-Ke Wang[1,2], Nan Jiang[1,2], and Zheng-Hong Lu[1,2,3] **

[1]*Department of Physics, Yunnan University, Kunming, Yunnan 650091, People's Republic of China*

[2]*Yunnan Key Laboratory for Micro/Nano Materials and Technology, Yunnan University, Kunming, Yunnan 650091, People's Republic of China*

[3]*Department of Materials Science and Engineering, University of Toronto, Toronto, Ontario M5S 3E4, Canada*

[*] Corresponding author: zhenghong.lu@utoronto.ca (Z.H. Lu)

Two types of transfer matrix are used in calculation: Transmission matrix L, which indicates the phase change in the same layer and is related to the thickness of material and to the angle between the surface normal and the wave vector; Interface matrix I, which indicates the reflection and refraction at interface of two media and is obtained by Fresnel reflection and transmission coefficients. Optical electric field E coming out of the OLED can be calculated by the methods descripted in reference[1].

To investigate the weak microcavity effect in OLED devices, microcavity effect factor is introduced[2] in calculation. The output optical electric field E_{out} can be described by equation (1):

$$E_{out} = \sqrt{\frac{[1+R_t+2\sqrt{R_t}\cos(-\varphi_t+\frac{4\pi n_{source} d cos(\theta)}{\lambda}}{(1-\sqrt{R_t R_b})^2+4\sqrt{R_t R_b} sin^2(\frac{\Delta\varphi}{2})}} E \qquad (1)$$

R_t and R_b are the reflectivity of cathode and anode respectively, n_{source} is the refractive index of emitting layer, and d is the distance between dipole source and cathode. $\Delta\varphi$ is the total phase shift in the cavity, φ_t is the phase shift at cathode. So, the relative spectral power distribution $P(\lambda)$ is calculated by:

$$P(\lambda) = |E_{out}|^2 \qquad (2)$$

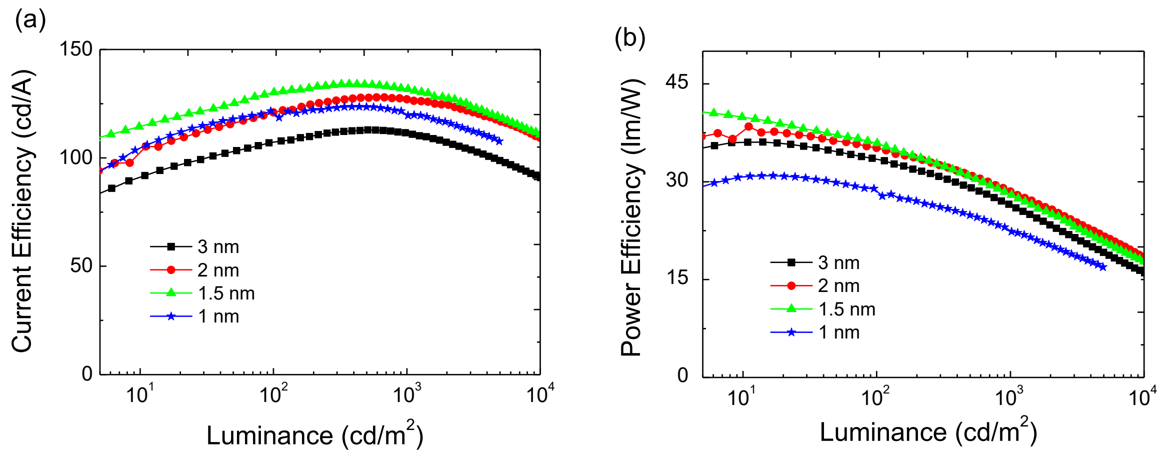

Figure S1. (a) Current efficiency–luminance, (b) Power efficiency–luminance characteristics of devices with various thicknesses of Al layer in CGL based on optical modeling. The structure of device is ITO/MoO_3(1 nm)/CBP(20 nm)/CBP:Ir(ppy)$_2$(acac)(8 wt.%,30 nm)/TPBi(65 nm)/Liq(1 nm)/Al(x nm)/MoO_3(10 nm)/CBP(20 nm)/CBP:Ir(ppy)$_2$(acac)(8 wt.%,30 nm)/TPBi (65 nm)/LiF(1 nm)/Al(100 nm).

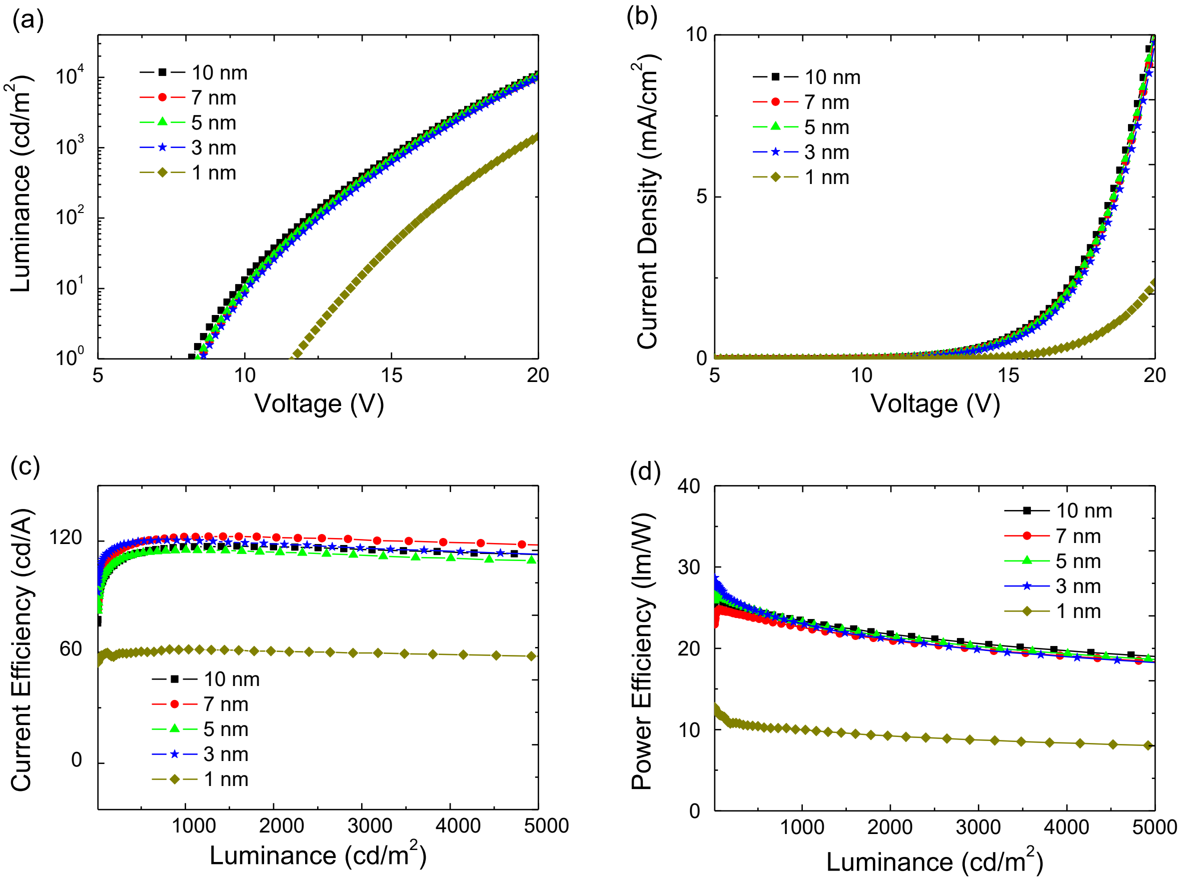

Figure S2. (a) Luminance–voltage (L-V), (b) Current density–voltage (J-V), (c) Current efficiency–luminance, (d) Power efficiency–luminance characteristics of devices with various thicknesses of MoO_3 layer in CGL. The structure of device is ITO/MoO_3(1 nm)/CBP(20 nm)/CBP:Ir(ppy)$_2$(acac)(8 wt.%,30 nm)/TPBi(65 nm)/Liq(1 nm)/Al(2 nm)/MoO_3(x nm)/CBP(20 nm)/CBP:Ir(ppy)$_2$(acac)(8 wt.%,30 nm)/TPBi (65 nm)/LiF(1 nm)/Al(100 nm).

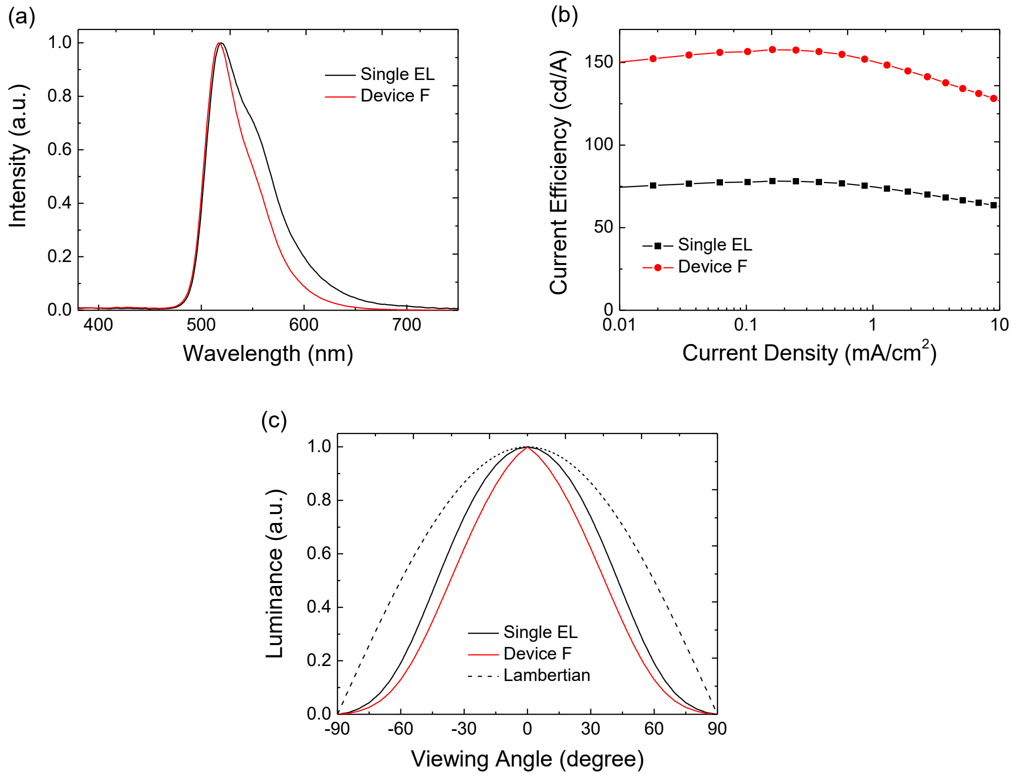

Figure S3. (a) Calculated spectral radiance, (b) Calculated Current efficiency–current density, (c) Simulated angular distribution of the luminance (normalized to 0˚ intensity) for the single EL unit device and tandem device F. The dashed line indicates Lambertian distribution.

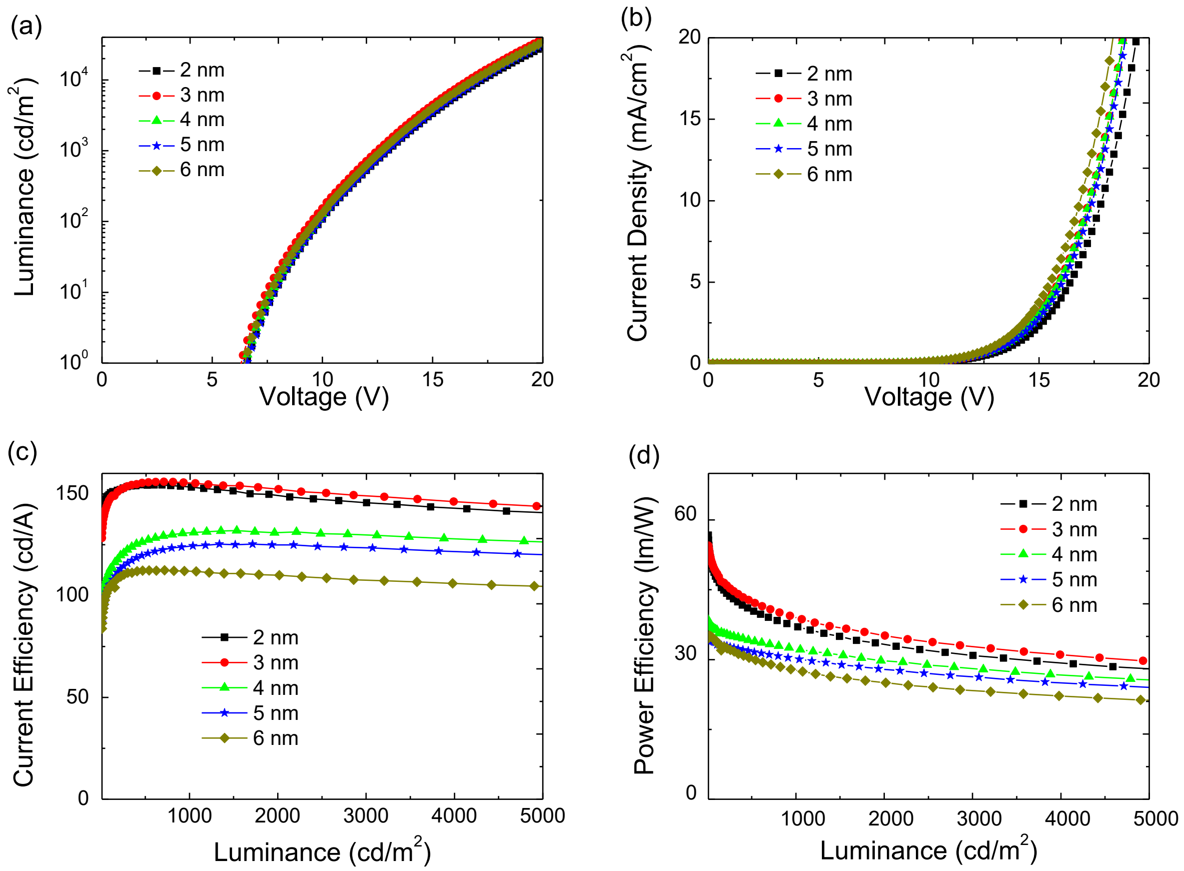

Figure S4. (a) Luminance–voltage (L-V), (b) Current density–voltage (J-V), (c) Current efficiency–luminance, (d) Power efficiency–luminance characteristics of devices with various thicknesses of Al layer in CGL. The structure of device is ITO/MoO_3(1 nm)/CBP(20 nm)/CBP:Ir(ppy)$_2$(acac)(8 wt.%,30 nm)/TPBi(65 nm)/Liq(1 nm)/Al(x nm)/C60(2 nm)/CBP:MoO_3(50 wt.%,10 nm)/CBP(20 nm)/CBP:Ir(ppy)$_2$(acac)(8 wt.%,30 nm)/TPBi(65 nm)/LiF(1 nm)/Al(100 nm).

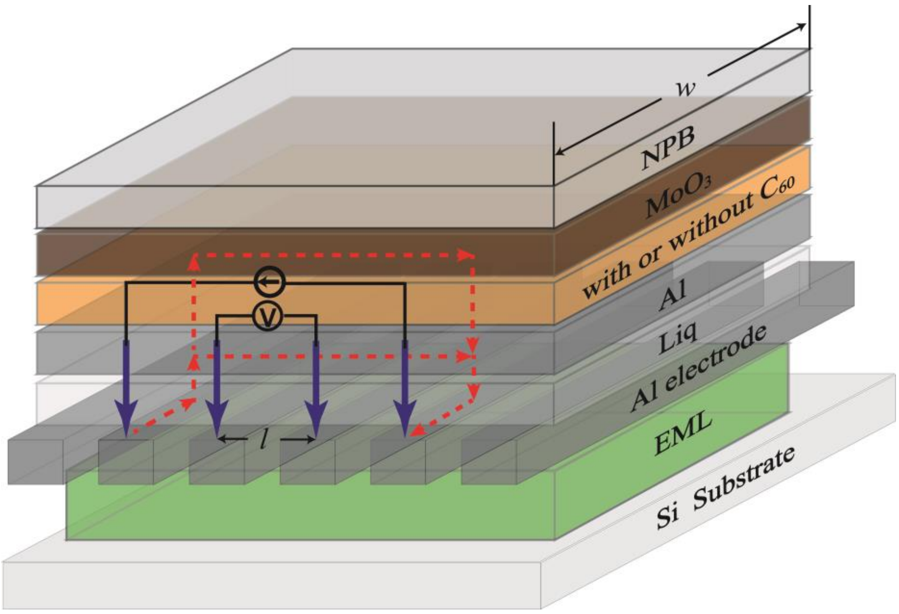

Figure S5. The schematic diagram of a test structure for measuring connecting electrode lateral resistance. The red dotted lines with arrows denote the current direction in the working structure.

To elucidate the impact of lateral conductivity by inserting C_{60} between Al and MoO_3 layers on CGL, we investigated the lateral conductivity of the devices [with a test structure: Si substrate/ CBP(20 nm)/ CBP:Ir(ppy)$_2$(acac) (8 wt.%, 30 nm)/ TPBi(65 nm)/ Al(100 nm)/ Liq(1 nm)/Al(2 nm)/with or without C_{60}(2 nm)/ MoO_3(10 nm)/NPB(50 nm)]. The total lateral resistance on CGL was measured by a four point probe measurement technique at ambient temperature. The schematic diagram of the test structure for measuring connecting electrode lateral resistance is shown in Figure S5. The measurement technique uses four finger-electrodes where the outer two electrodes supply a constant current and the voltage drop over the two inner electrodes is measured. The spacing of these finger electrodes is 1 mm.

The total resistance *R* of the CGL can be calculated by the Ohm's law:

$$R = {}^{V_{measured}}/_{I_{measured}} \quad (3)$$

where $V_{measured}$ and $I_{measured}$ are the measured electric potential and the measured current.

The lateral resistivity ρ can be calculated using:

$$\rho = {R \cdot d \cdot w}/{l} \qquad (4)$$

where w and d are the width and the thickness of the CGL layer, l is the spacing between the two inner electrodes. Values of the lateral electric conductivity σ were obtained by simply inverting the corresponding values of the lateral resistivity as follows:

$$\sigma = \rho^{-1} \qquad (5)$$

In our test structure, the width w of the films is 16 mm and the electrode spacing l is 1 mm. The measured electric potential V of the CGL films without/with a C_{60} layer is 44.25 mV and 57.03 mV at a constant current of 4.459 μA and 0.4459 μA, respectively. Due to the reduction reaction between Al and MoO_3 leading to formation of a laterally conducting interfacial MoO_{3-x} layer, the thickness of the lateral conducted layers could vary from 2 nm (just Al layer) to 12 nm (Al+MoO_3 layer). Thus the lateral electric conductivity of the CGL films without a C_{60} layer is in the range 5.25-31.50 S/cm, while the CGL lateral conductivity with a C_{60} layer is in the range 0.41-2.44 S/cm.

1 Wang, Z. B. *et al.* Optical design of organic light emitting diodes. *J. Appl. Phys.* **109**, 053107 (2011).

2 Thomschke, M., Nitsche, R., Furno, M. & Leo, K. Optimized efficiency and angular emission characteristics of white top-emitting organic electroluminescent diodes. *Appl. Phys. Lett.* **94**, 083303 (2009).
